# NGL-1/LRRC4C Deletion Moderately Suppresses Hippocampal Excitatory Synapse Development and Function in an Input-Independent Manner

**DOI:** 10.3389/fnmol.2019.00119

**Published:** 2019-05-14

**Authors:** Yeonsoo Choi, Haram Park, Hwajin Jung, Hanseul Kweon, Seoyeong Kim, Soo Yeon Lee, Hyemin Han, Yisul Cho, Seyeon Kim, Woong Seob Sim, Jeongmin Kim, Yongchul Bae, Eunjoon Kim

**Affiliations:** ^1^Department of Biological Sciences, Korea Advanced Institute for Science and Technology (KAIST), Daejeon, South Korea; ^2^Center for Synaptic Brain Dysfunctions, Institute for Basic Science (IBS), Daejeon, South Korea; ^3^Department of Anatomy and Neurobiology, School of Dentistry, Kyungpook National University, Daegu, South Korea

**Keywords:** synapse, trans-synaptic adhesion, NGL-1, LRRC4C, PSD-95, synaptic transmission, synaptic plasticity

## Abstract

Netrin-G ligand-1 (NGL-1), also known as LRRC4C, is a postsynaptic densities (PSDs)-95-interacting postsynaptic adhesion molecule that interacts trans-synaptically with presynaptic netrin-G1. NGL-1 and its family member protein NGL-2 are thought to promote excitatory synapse development through largely non-overlapping neuronal pathways. While NGL-2 is critical for excitatory synapse development in specific dendritic segments of neurons in an input-specific manner, whether NGL-1 has similar functions is unclear. Here, we show that *Lrrc4c* deletion in male mice moderately suppresses excitatory synapse development and function, but surprisingly, does so in an input-independent manner. While NGL-1 is mainly detected in the stratum lacunosum moleculare (SLM) layer of the hippocampus relative to the stratum radiatum (SR) layer, NGL-1 deletion leads to decreases in the number of PSDs in both SLM and SR layers in the ventral hippocampus. In addition, both SLM and SR excitatory synapses display suppressed short-term synaptic plasticity in the ventral hippocampus. These morphological and functional changes are either absent or modest in the dorsal hippocampus. The input-independent synaptic changes induced by *Lrrc4c* deletion involve abnormal translocation of NGL-2 from the SR to SLM layer. These results suggest that *Lrrc4c* deletion moderately suppresses hippocampal excitatory synapse development and function in an input-independent manner.

## Introduction

Synaptic adhesion molecules regulate neuronal synapse development and synaptic transmission and plasticity (Shen and Scheiffele, 2010; Yuzaki, 2011; Krueger et al., 2012; Missler et al., 2012; Valnegri et al., 2012; Takahashi and Craig, 2013; Um and Ko, 2013; Bemben et al., 2015; Ko et al., 2015; de Wit and Ghosh, 2016; Sudhof, 2017, 2018; Um and Ko, 2017). In addition, deficits in synaptic adhesion molecules can disrupt normal development of neuronal circuits, leading to abnormal brain functions and behaviors (Sudhof, 2008; Valnegri et al., 2012; Ko et al., 2015).

Netrin-G ligand (NGL) proteins (also known as LRRC4) are a family of postsynaptic adhesion molecules with the known members, NGL-1/LRRC4C, NGL-2/LRRC4 and NGL-3/LRRC4B (Lin et al., 2003; Kim et al., 2006; Woo et al., 2009b). NGLs are mainly expressed in the brain, although NGL-1 and NGL-3 mRNAs are also detected in liver and heart, respectively (Lin et al., 2003; Zhang et al., 2005; Kim et al., 2006). NGLs contain extracellular adhesion domains followed by a single transmembrane domain and a cytoplasmic region ending with a C-terminal PDZ domain-binding motif that directly binds to postsynaptic densities (PSDs)-95, an abundant PDZ-containing postsynaptic scaffolding protein (Sheng and Sala, 2001; Sheng and Hoogenraad, 2007; Sheng and Kim, 2011). NGL-1, NGL-2, and NGL-3 also bind to the specific presynaptic adhesion molecules, netrin-G1, netrin-G2, and LAR family receptor tyrosine phosphatases (LAR, PTPσ, and PTPδ), respectively (Nakashiba et al., 2000, 2002; Yin et al., 2002; Lin et al., 2003; Kim et al., 2006; Woo et al., 2009a; Kwon et al., 2010; Seiradake et al., 2011). These trans-synaptic interactions have been shown to contribute to aspects of synapse development and function (Nishimura-Akiyoshi et al., 2007; Woo et al., 2009a; Kwon et al., 2010; DeNardo et al., 2012; Song et al., 2013; Matsukawa et al., 2014; Um et al., 2018). For instance, postsynaptic NGL-3 interacts with presynaptic LAR family receptor tyrosine phosphatases to promote pre- and postsynaptic development in a synapse-formation assay (Woo et al., 2009a; Kwon et al., 2010).

Intriguingly, NGL-1 and NGL-2 are distributed to specific dendritic segments in the same neuron that receive inputs from different axons. Specifically, NGL-1 is distributed to the distal dendrites of CA1 pyramidal neurons in the stratum lacunosum moleculare (SLM) layer of the hippocampus that receive synaptic inputs from entorhinal cortex (EC) layer III neurons through the temporoammonic-CA1 neuronal (TA-CA1) pathway, whereas NGL-2 is mainly distributed to the proximal dendrites of CA1 pyramidal neurons in the stratum radiatum (SR) layer that receives inputs from CA3 pyramidal neurons through the Schaffer collateral-CA1 (SC-CA1) pathway (Niimi et al., 2007; Nishimura-Akiyoshi et al., 2007). However, whether NGL-3 displays a similar laminar-specific localization remains unclear.

Functionally, *Lrrc4* (or NGL-2) deletion in mice suppresses excitatory synapse development and synaptic transmission in SC-CA1 synapses in an input-specific manner with synaptic changes occurring specifically in the NGL-2-enriched SC-CA1 pathway but not in the NGL-2-non-enriched TA-CA1 pathway (DeNardo et al., 2012; Um et al., 2018), although a different result (no effect) has also been reported (Matsukawa et al., 2014). However, apart from a reported effect on post-tetanic potentiation (PTP) in the TA-CA1 pathway (Matsukawa et al., 2014), whether deletion of *Lrrc4c* (or NGL-1) in mice leads to similar input-specific deficits in synapse development and function, for instance, in the TA-CA1 pathway relative to the SC-CA1 pathway, remains unclear.

In the present study, we examined the impacts of *Lrrc4c* deletion on hippocampal synapses in mice and found that *Lrrc4c* deletion modestly suppresses hippocampal synapse development and function in an input-independent manner with synaptic changes occurring in both NGL-1-enriched TA-CA1 and NGL-1-non-enriched SC-CA pathways.

## Materials and Methods

### Animals

The *Lrrc4c^−/−^* mice used in this study have been previously described (Um et al., 2018). Briefly, *Lrrc4c*^−/−^ mice (LRRC4C^tm1Lex^), obtained from The Mutant *Mouse* Resource and Research Center, were generated by introducing an NGL-1 targeting vector into 129/SvEvBrd-derived embryonic stem (ES) cells by homologous recombination, thereby replacing the third exon of the *Lrrc4c* gene encoding NGL-1 with a β-geo (LacZ/neo) cassette. These mice were mated with C57BL/6J albino mice, and the resulting F1 heterozygous mice were crossed with C57BL/6J mice for more than five generations to obtain *Lrrc4c*^−/−^ mice in a C57BL/6J background. Mice were weaned at postnatal day 21, and mixed-genotype littermates of the same sex were housed together until experiments. All animals were fed *ad libitum* and housed under a 12-h light/dark cycle (light phase from 1:00 to 13:00). Mouse maintenance and procedures were performed in accordance with the Requirements of Animal Research at KAIST, and all procedures were approved by the Committee of Animal Research at KAIST (KA2012-19). For genotyping, the following primers were used. WT-for: GAACAAGATGACCTTACATCC, WT-rev: CAATAGGGTTGTTCCTCAACCAG, mut-for: CCCTAGGAATGCTCGTCAAGA, and mut-rev: CAGACTGTTTGAACTCCAGAAG (WT band size: 476 base pairs, mut band size: 289 base pairs).

### LacZ Staining

Mice were deeply anesthetized with isoflurane before the procedure. Brains were isolated from male mice (3 weeks old) after cardiac perfusion (4% paraformaldehyde or PFA). After post-fixation for 2 h, brain sections (250 μm) were obtained by vibratome (VT1200s, Leica). Brain sections were washed three times with 1× phosphate buffered saline (PBS) for 10 min and immersed in X-gal staining buffer (5 mM potassium hexacyanoferrate III (K3), 5 mM potassium hexacyanoferrate II trihydrate (K4), 2 mM MgCl_2_, 0.01% deoxycholate, 0.02% NP40, and 1 mg/ml X-gal in 1× PBS) for 3–5 h at room temperature. During the staining, the sample was kept out from the light since X-gal is light-sensitive. After staining, it was washed thoroughly for 10 min five times. Before the mounting with Vectashield (Vectorlabs), the sample was fixated for 30 min and washed again for 5 min with 1× PBS. Imaging was performed by light microscopy.

### Electron Microscopy

WT and *Lrrc4c^−/−^* mice were deeply anesthetized with sodium pentobarbital (80 mg/kg, i.p.) and were intracardially perfused with 10 ml of heparinized normal saline, followed by 50 ml of a freshly prepared fixative of 2.5% glutaraldehyde and 1% PFA in 0.1 M phosphate buffer (PB, pH 7.4). Hippocampus was removed from the whole brain, postfixed in the same fixative for 2 h and stored in PB (0.1 M, pH 7.4) overnight at 4°C. Sections were cut coronally or horizontally on a Vibratome at 50 μm. The sections were osmicated with 1% osmium tetroxide (in 0.1 MPB) for 1 h, dehydrated in graded alcohols, flat embedded in Durcupan ACM (Fluka), and cured for 48 h at 60°C. Small pieces containing SR and stratum lacunosum-moleculare (SLM) of dorsal and ventral hippocampal CA1 regions were cut out of the wafers and glued onto the plastic block by cyanoacrylate. We dissected out the hippocampus and cut it in half in the middle of the septotemporal axis to obtain the dorsal and ventral hippocampus. We then used the middle region of the dorsal or ventral hippocampus for electron microscopy (EM) analysis. Ultrathin sections were cut and mounted on Formvar-coated single-slot grids. For quantification of excitatory synapse, sections were stained with uranyl acetate and lead citrate, and examined with an electron microscope (Hitachi H-7500; Hitachi) at 80 kV accelerating voltage. Twenty-four micrographs representing 368.9 μm^2^ neuropil regions in each mouse were photomicrographed at a 40,000× and used for quantification. Number of spines (PSD density), proportion of perforated spines, PSD length and PSD thickness from three WT and *Lrrc4c*^−/−^ mice were quantified. The measurements were all performed by an experimenter blind to the genotype. Digital images were captured with GATAN DigitalMicrograph software driving a CCD camera (SC1000 Orius; Gatan) and saved as TIFF files. Brightness and contrast of the images were adjusted in Adobe Photoshop 7.0 (Adobe Systems).

### Immunoblot Analysis

Whole-brain were homogenized by motorized tissue grinder in ice-cold homogenization buffer (0.32 M sucrose, 10 mM HEPES, 2 mM EDTA, 2 mM EGTA containing protease and phosphatase inhibitors). For subcellular fractionations, the homogenates were centrifuged at 900 *g* for 10 min (the resulting pellets are P1). The resulting supernatants were centrifuged again at 12,000 *g* for 15 min (the supernatants are S2). The pellets were resuspended in homogenization buffer and centrifuged at 13,000 *g* for 15 min (the resulting pellets are P2 or crude synaptosomes). Each fraction was lysed in 2× SDS lysis buffer and boiled for 15 min.

For microdissection of SR and SLM samples, ventral hippocampal CA1 sections (400 μm) were sliced in sCSF with vibratome (VT1200s, Leica). Under light microscopy, SR and SLM regions, which are readily discernable by their different darkness (SLM is darker), were manually dissected on an ice-cold platform, and four animals were pooled for one sample. Each sample was homogenized in 60–80 μl of ice-cold homogenization buffer and the whole lysates were used without any further fractionation. Total of 32 of adult (2–3 months) male mice were used to make *n* number of eight pairs by pooling four pairs of mice. The band was analyzed using Odyssey imaging program.

### Antibodies

The following antibodies were previously described: NGL-1 (#2040), NGL-2 (#2044; Um et al., 2018), NGL-3 (#1948; Lee et al., 2014), GluA1 (#1193), GluA2 (#1195; Kim et al., 2009). In addition, the following antibodies were purchased from commercial sources: GluN1 (Neuromab 75-272), GluN2A (Alomone AGC-003), GluN2B (Neuromab 75-101), PSD-95 (Neuromab 75-028), protein kinase Cα (PKCα; BD transduction 610108), CaMKIIα/β (Cell signaling 3362) and α-tubulin (Sigma T5168).

### Brain Slices for Electrophysiology

Acute horizontal brain slices were obtained by anesthetizing 2–4-month-old adult male mice with isoflurane (Terrell, Piramal Critical Care) and extracting the brain into a 0°C dissection buffer consisting of, in mM: 212 sucrose, 25 NaHCO_3_, 5 KCl, 1.25 NaH_2_PO_4_, 10 D-glucose, 2 sodium pyruvate, 1.2 sodium ascorbate, 3.5 MgCl_2_, 0.5 CaCl_2_ and bubbled with 95% O_2_/5% CO_2_. The dorsal part of the brain was fixated with cyanoacrylate glue onto a triangular agar gel with a 12-degree angle and transferred to a vibratome (VT1200s, Leica), where horizontal brain sections were obtained (Remondes and Schuman, 2002). To obtain dorsal hippocampal sections, hemi-sectioned brains were glued along the midline surface. Brains were sliced at a thickness of 400 μm, and resulting slices were transferred to a 32°C holding chamber containing a solution of artificial cerebrospinal fluid (aCSF; in mM: 125 NaCl, 25 NaHCO_3_, 2.5 KCl, 1.25 NaH_2_PO_4_, 10 D-glucose, 1.3 MgCl_2_, 2.5 CaCl_2_). Slices were recovered at 32°C for 1 h, and afterward further recovered in room temperature (20–25°C) for 30 min. Once recovery was finished, slices were transferred to a recording chamber, where all electrophysiological experiments were performed at 28°C with circulating aCSF. Cells were visualized under differential interference contrast illumination in an upright microscope (B50WI, Olympus).

### Whole-Cell Recording

For whole-cell voltage-clamp recordings, thin-walled borosilicate capillaries (30-0065, Harvard Apparatus) were used to make pipettes with resistance 2.3–3.5 MΩ *via* a two-step vertical puller (PC-10, Narishige). For miniature excitatory postsynaptic current (mEPSC) recordings, pipettes were filled with an internal solution composed of, in mM: 117 CsMeSO_4_, 10 EGTA, 8 NaCl, 10 TEACl, 10 HEPES, 4 Mg-ATP, 0.3 Na-GTP, 5 QX-314. For miniature inhibitory postsynaptic current (mIPSC) recordings, the internal solution contained, in mM: 115 CsCl, 10 EGTA, 8 NaCl, 10 TEACl, 10 HEPES, 4 Mg-ATP, 0.3 Na-GTP, 5 QX-314. For the measurement of intrinsic neuronal properties, pipettes were filled with an internal solution containing, in mM, 137 potassium gluconate, 5 KCl, 10 HEPES, 0.2 EGTA, 10 sodium phosphocreatine, 4 Mg-ATP, 0.5 Na-GTP. All internal solutions were titrated to pH 7.35 and adjusted to an osmolarity of 285 mOsm. For mEPSC experiments, 60 μM picrotoxin and 0.5 μM tetrodotoxin (Tocris) were added to the aCSF. For mIPSC experiments 10 μM NBQX (Tocris), 50 μM D-AP5 (Tocris), 0.5 μM tetrodotoxin (Tocris) were added. For neuronal property experiments, 10 μM NBQX (Tocris), 50 μM D-AP5 (Tocris), and 60 μM picrotoxin. Access resistance was maintained as to be no greater than 20 MΩ or else excluded from data acquisition. Signals were filtered at 2 kHz and digitized at 10 kHz under the control of Multiclamp 700B Amplifier (Molecular Devices) and Digidata 1550 Digitizer (Molecular Devices). Cells were approached with the internal solution-filled pipette to make a giga seal, after which cells were gently ruptured *via* suction and maintained thereafter at −70 mV. After voltage-clamped cells were stabilized (~3 min post-rupture), recordings were obtained. Access resistance was monitored throughout the stabilization period and immediately before and after the data acquisition. The acquired data were analyzed and extracted using Clampfit 10 (Molecular Devices).

### Field Recording

For field EPSP (fEPSP) recordings, baseline responses were collected at 0.07 Hz with a stimulation intensity that yielded a half-maximal response. Once a stable baseline response was acquired, excitatory transmissions were evoked at a set series of increasing stimuli using an isolated pulse stimulator (A-M Systems). fEPSP slopes and fiber volleys were then interpolated *via* linear fits to obtain an input/output model of basal evoked excitatory transmissions. For paired-pulse facilitation, pairs of peak amplitude responses were obtained at indicated inter-pulse intervals and divided to obtain the PPF ratios for each inter-pulse interval. For release probability, a single 20 Hz, 5-s stimulation was given in the presence of 2 μM kynurenic acid (Tocris), and 0.05 μM cyclothiazide (Tocris). Signals were filtered at 2 kHz and digitized at 1 kHz under control of Multiclamp 700B Amplifier and Digidata 1550 Digitizer. The acquired data were analyzed using Clampfit 10. theta burst stimulation (TBS)-long-term potentiation (LTP) was induced by four episodes of TBS with 10 s intervals. TBS consisted of 10 stimulus trains delivered at 5 Hz; each train consisted of four pulses at 100 Hz. High-frequency stimulation (HFS)-LTP was induced by a single delivery of a 100 Hz, 1-s stimulus. Average responses (±SEM) are expressed as percentages of baseline responses.

### Experimental Design and Statistical Analysis

All quantitative analyses were performed using age-matched WT (C57BL/6J) and *Lrrc4c^−/−^* mice. For EM, six frames were imaged for each animal, and each frame was taken as an “n” of 1. For electrophysiological analyses, three to five animals were used in each experiment. Normality and equal variance were tested for all statistical analysis using D’Agostino-Pearson omnibus normality test. Sample size was determined by the nature of the experimental design. Statistical tests were performed using Prism 5 (GraphPad) and SigmaPlot 12.0 (Systat Software). Two-way analysis of variance (ANOVA) was performed for tests of both genotype and any additional factors; Holm-Sidak multiple comparison *post hoc* analyses were performed only when either the interaction or both main factors were significant.

## Results

### Generation and Basic Characterization of *Lrrc4c*^−/−^ Mice

**Figure 1:**
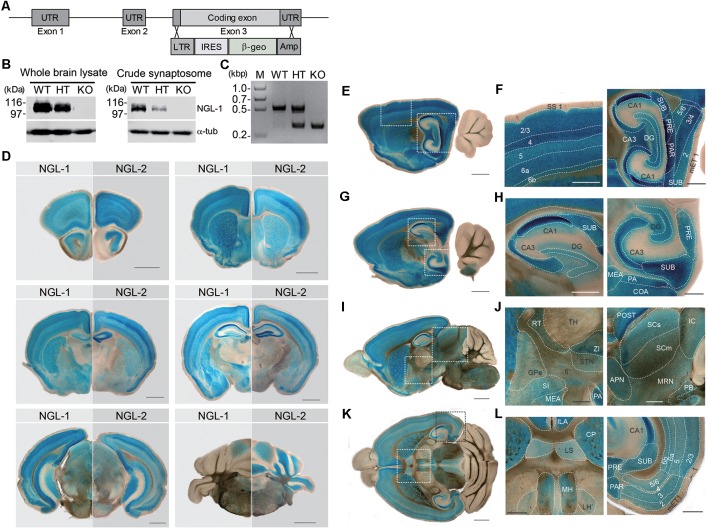
Generation and basic characterization of *Lrrc4c^−/−^* mice. **(A)** Schematic depiction of the *Lrrc4c* knockout (KO) strategy. UTR, untranslated region; LTR, long terminal repeat; IRES, internal ribosome entry site; β-geo, a gene encoding the fusion of β-galactosidase and neomycin; Amp, ampicillin resistance gene. **(B)** Netrin-G ligand-1 (NGL-1) protein levels in the brains of WT and heterozygous (HT; *Lrrc4c*^+/−^) and homozygous (KO; *Lrrc4c*^−/−^) NGL-1 mutant mice, shown by immunoblot analysis of whole-brain lysates or crude synaptosomes using NGL-1 antibodies (#2040). **(C)** Polymerase chain reaction (PCR) genotyping of WT and *Lrrc4c*–HT and –KO mice. **(D)** Comparison of NGL-1 and NGL-2 expression patterns by X-gal staining of coronal brain slices of heterozygous mutant NGL-1 (*Lrrc4c*^+/−^) and NGL-2 (*Lrrc4*^+/−^) mice (3 weeks; male). Scale bar, 1 mm. **(E–L)** X-gal–stained sagittal **(E–J)** and horizontal **(K,L)** *Lrrc4c*^+/−^ brain sections (3 weeks; male). Dotted square areas in **(E,G,I,K)** are enlarged to show NGL-1 expression patterns in detail [**F**, enlarged images of somatosensory cortex and hippocampus; **H**, dorsal and ventral hippocampus; **J**, thalamus, striatum and hypothalamus, and midbrain; **L**, habenula and entorhinal cortex (EC)]. Abbreviations; anterior pretectal nucleus (APN), cornu ammonus 1 (CA1), cornu ammonus 3 (CA3), cortical amygdalar area (COA), caudoputamen (CP), dentate gyrus (DG), medial entorhinal cortex (mET), fiber tract (fi), globus pallidus, external segment (GPe), inferior colliculus (IC), infralimbic area (ILA), lateral habenula (LH), lateral septum (LS), medial amygdalar nucleus (MEA), medial habenula (MH), midbrain reticular nucleus (MRN), posterial amygdalar nucleus (PA), parasubiculum (PAR), parabigeminal nucleus (PB), presubiculum (PRE), postsubiculum (POST), reticular nuclues of thalamus (RT), suprior colliculus, sensory related (SCs), superior colliculus, motor related (SCm), substantia innominata (SI), somatosensory cortex (SS), subiculum (SUB), subthalamic nucleus (STN), thalamus (TH), zona incerta (ZI), and cortical layer numbers (numbers). Scale bar, 0.5 mm.

To understand *in vivo* functions of NGL-1, we generated *Lrrc4c*–knockout (KO) mice (*Lrrc4c*^−/−^ mice) by replacing exon 3 of the *Lrrc4c* gene encoding the entire NGL-1 protein with a β-geo cassette through homologous recombination (Figure 1A). KO of NGL-1 in *Lrrc4c*^−/−^ mice was validated at the DNA and protein level by polymerase chain reaction (PCR) and Western blotting, respectively (Figures 1B,C).

Because the coding region was replaced with a β-geo cassette expressing β-galactosidase, the putative expression pattern of the NGL-1 protein in the mouse brain could be observed by X-gal staining without the requirement for specific antibodies, which often cross-react with other antigens. For comparison, parallel experiments were performed using another mouse line in which the β-geo cassette replaced the NGL-2 (*Lrrc4*) gene (Um et al., 2018).

NGL-1 and NGL-2 signals were detected in various overlapping and distinct mouse brain regions. The overlapping regions included the cortex, hippocampus, striatum, and amygdala (Figure 1D). NGL-1 signals were stronger than those of NGL-2 in certain regions, including the thalamic reticular nucleus (TRN), hypothalamus and habenula, whereas NGL-2 signals were stronger in the cerebellum.

Enlarged images showed that NGL-1 signals were particularly strong in layer 4 of the somatosensory cortex (Figures 1E,F). In the hippocampus, NGL-1 signals were stronger in the CA1 region relative to other subregions of the dorsal and ventral hippocampus (Figures 1G,H). NGL-1 signals were also detected in other brain regions, including the medial habenula (MH), superior and inferior colliculus (SC and IC), and lateral septum (LS; Figures 1I–L).

### Decreased PSD Density in SR and SLM Layers of the *Lrrc4c*^−/−^ Ventral CA1 Region

**Figure 2:**
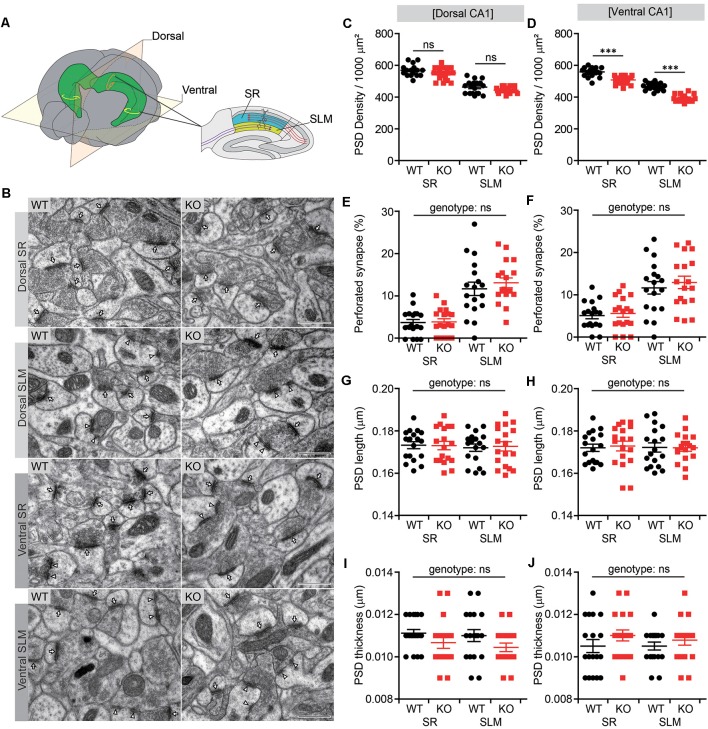
Decreased number of postsynaptic densities (PSDs) in both SR and SLM layers of the ventral CA1 region in *Lrrc4c^−/−^* mice. **(A)** Diagram of examined hippocampal regions. Dorsal and ventral, dorsal and ventral regions of the hippocampus; SR and SLM, stratum radiatum and stratum lacunosum-moleculare regions in the CA1 region. **(B)** Representative images of electron microscopy (EM) data for dorsal SR, dorsal SLM, ventral SR and ventral SLM layers of the hippocampal CA1 region of WT and *Lrrc4c*^−/−^ mice (2 months; male). Arrows indicates PSD in non-perforated synapses, and arrowheads indicate PSD in perforated synapses. Scale bar 500 nm. **(C–J)** Density, length, thickness, and perforation of PSDs apposed to presynaptic axon terminals. Note that the density of the PSD is modestly decreased in the ventral, but not dorsal, CA1 region. *n* = 18 sections from three mice (WT), *n* = 18 (3; KO), ns, not significant, two-way analysis of variance (ANOVA) with Holm-Sidak multiple comparison test (**C**: *F*_(1,68)_ = 0.0119, *p* = 0.9137; genotype, *F*_(1,68)_ = 4.74, *p* = 0.0329; layer, *F*_(1,68)_ = 165, *p* < 0.001; SR, *p* = 0.2090, SLM, *p* = 0.2090; **D**: interaction, *F*_(1,68)_ = 4.0813, *p* = 0.0473; genotype, *F*_(1,68)_ = 108.16, *p* < 0.001; layer, *F*_(1,68)_ = 288.45, *p* < 0.001; SR, ***p < 0.001, SLM, ***p < 0.001); **E**: interaction, *F*_(1,68)_ = 0.5163, *p* = 0.4749; genotype, *F*_(1,68)_ = 0.3255, *p* = 0.5702; layer, *F*_(1,68)_ = 58.06, *p* < 0.001; **F**: interaction, *F*_(1,68)_ = 0.1037, *p* = 0.7484; genotype, *F*_(1,68)_ = 0.5725, *p* = 0.4519; layer, *F*_(1,68)_ = 33.9, *p* < 0.001; **G**: interaction, *F*_(1,68)_ = 0.04169, *p* = 0.8388; genotype, *F*_(1,68)_ = 0.01361, *p* = 0.9075; layer, *F*_(1,68)_ = 0.1668, *p* = 0.6843; **H**: interaction, *F*_(1,68)_ = 0.06493, *p* = 0.7996; genotype, *F*_(1,68)_ = 0.02886, *p* = 0.8656; layer, *F*_(1,68)_ = 0.02886, *p* = 0.8656; **I**: interaction, *F*_(1,68)_ = 0.05537, *p* = 0.8147; genotype, *F*_(1,68)_ = 4.485, *p* = 0.0378; layer; *F*_(1,68)_ = 0.4984, *p* = 0.4826; **J**: interaction, *F*_(1,68)_ = 0.1985, *p* = 0.6573; genotype, *F*_(1,68)_ = 2.432, *p* = 0.1235; layer, *F*_(1,68)_ = 0.1985, *p* = 0.6573).

It has been shown that NGL-1 expressed in CA1 neurons is targeted to and maintained at the SLM layer through trans-synaptic interactions with presynaptic netrin-G1 (Nishimura-Akiyoshi et al., 2007). Conversely, presynaptic localization of netrin-G1 has been shown to require postsynaptic NGL-1 (Matsukawa et al., 2014). These results suggest that the trans-synaptic interaction between NGL-1 and netrin-G1 is important for their synaptic localization in the SLM as well as excitatory synapse development and function in this region. We, therefore, tested whether *Lrrc4c* deletion affected the density of excitatory synapses by analyzing PSDs apposed to presynaptic axon terminals in both SR and SLM layers of the CA1 region using EM (Figure 2A).

There were moderate decreases in the density of PSDs in both SR and SLM layers of the ventral CA1 region (Figures 2B,D). Similar trends were observed in the dorsal CA1 region, but these changes did not reach statistical significance (Figure 2C). Other parameters of the PSD, such as PSD perforation (measure of maturation), length and thickness, in SR or SLM layers of dorsal or ventral CA1 regions were not different between genotypes, although the percentage of perforated PSDs was greater in the SLM layer relative to the SR layer (Figures 2E–J), as previously reported (Nicholson et al., 2006). These results suggest that *Lrrc4c* deletion leads to a moderate reduction in the number of excitatory synapses in both SR and SLM layers of the ventral, but not dorsal, CA1 region.

### Normal Spontaneous and Basal Synaptic Transmission in the *Lrrc4c*^−/−^ Hippocampus

To determine if the moderate decrease in PSD density in the ventral hippocampus of *Lrrc4c^−/−^* mice influences synaptic transmission, we first measured spontaneous synaptic transmission in CA1 pyramidal neurons in the morphologically altered ventral hippocampus.

**Figure 3:**
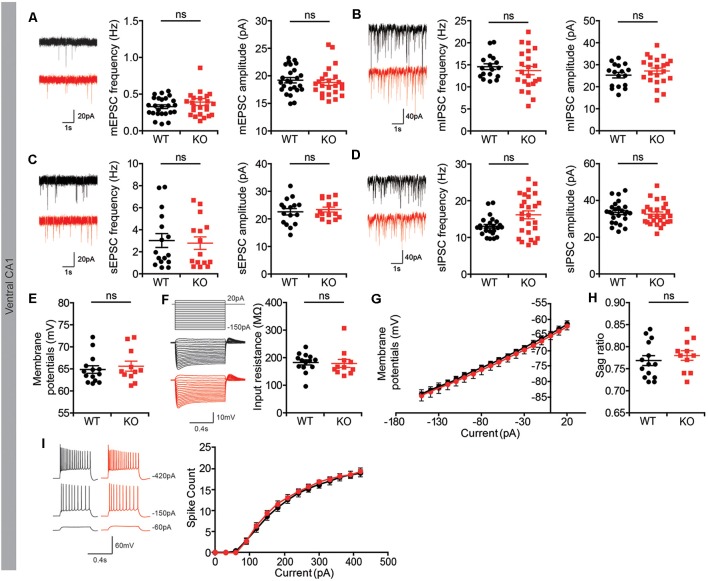
Normal spontaneous synaptic transmission in *Lrrc4c^−/−^* hippocampal CA1 pyramidal neurons. **(A)** Normal frequency and amplitude of miniature excitatory postsynaptic currents (mEPSCs) in ventral CA1 pyramidal neurons of *Lrrc4c*^−/−^ mice (2–4 months; male). *n* = 25 neurons from four mice (WT), *n* = 24 (4; KO), ns, not significant, Mann-Whitney test. (mEPSC frequency: *U* = 279.5; *p* = 0.8740; mEPSC amplitude: *U* = 261.0; *p* = 0.4447). **(B)** Normal frequency and amplitude of miniature inhibitory postsynaptic currents (mIPSCs) in ventral CA1 pyramidal neurons of *Lrrc4c*^−/−^ mice (2–4 months; male). *n* = 17 (4; WT), *n* = 22 (4; KO), ns, not significant, Student’s *t*-test. (mIPSC frequency: *t*_(37)_ = 0.7133; *p* = 0.4801; mIPSC amplitude: *t*_(37)_ = 1.028; *p* = 0.3107). **(C)** Normal frequency and amplitude of spontaneous EPSC (sEPSC) in ventral CA1 pyramidal neurons of *Lrrc4c*^−/−^ mice (2–4 months; male). *n* = 16 (3; WT), *n* = 15 (3; KO), ns, not significant, Student’s *t*-test. (sEPSC frequency: *t*_(29)_ = 0.28; *p* = 0.7815; sEPSC amplitude: *t*_(29)_ = 0.5433; *p* = 0.5911). **(D)** Normal frequency and amplitude of spontaneous IPSCs (sIPSCs) in ventral CA1 pyramidal neurons of *Lrrc4c*^−/−^ mice (2–4 months; male). *n* = 31 (5; WT), *n* = 26 (5; KO), ns, not significant, Mann-Whitney test (frequency) and Student’s *t*-test (amplitude). (sIPSC frequency: *U* = 218; *p* = 0.0692; sIPSC amplitude: *t*_(49)_ = 0.5373; *p* = 0.5935). **(E–I)** Normal resting membrane potential **(E)**, input resistance **(F,G)**, sag ratio **(H)**, current firing curves **(I)** in ventral CA1 pyramidal neurons of *Lrrc4c*^−/−^ mice (2–4 months; male). *n* = 14 (3; WT), *n* = 11 (3; KO), ns, not significant, Mann-Whitney test, two-way repeated-measures ANOVA with Holm-Sidak multiple comparison test (**E**: *U* = 71; *p* = 0.7675; **F**: *U* = 55; *p* = 0.2441; **H**: *U* = 61; *p* = 0.3937).

We found that both the frequency and amplitude of mEPSCs and mIPSCs were normal in *Lrrc4c^−/−^* CA1 neurons compared with those in WT neurons (Figures 3A,B). In addition, spontaneous EPSCs and IPSCs (sEPSCs and sIPSCs), which reflect local circuit activities, were also unaffected (Figures 3C,D). Intrinsic properties were normal in *Lrrc4c*^−/−^ CA1 neurons, as shown by comparing resting membrane potential, input resistance, sag ratio, and current-firing curves in neurons from these mice with those of WT neurons (Figures 3E–I). These results suggest that *Lrrc4c* deletion does not induce changes in spontaneous synaptic transmission or intrinsic neuronal properties in ventral CA1 neurons.

**Figure 4:**
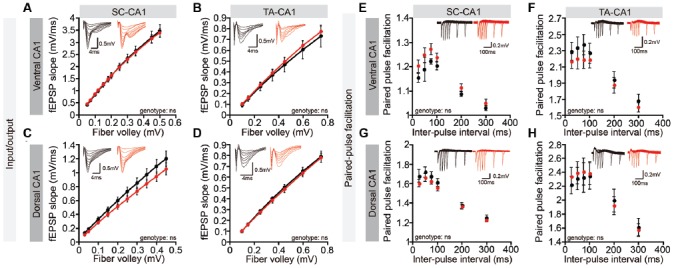
Normal basal excitatory synaptic transmission in the *Lrrc4c^−/−^* hippocampal CA1 region. **(A–D)** Normal basal excitatory synaptic transmission in the ventral SC-CA1 pathway **(A)**, ventral TA-CA1 pathway **(B)**, dorsal SC-CA1 pathway **(C)**, and dorsal TA-CA1 pathway **(D)** in the hippocampal CA1 region of *Lrrc4c*^−/−^ mice (2–4 months; male). Dorsal SC-CA1, *n* = 9 slices from three mice (WT), *n* = 10 (3; KO); dorsal TA-CA1, *n* = 8 (3; WT), *n* = 8 (3; KO); ventral SC-CA1, *n* = 12 (4; WT), *n* = 12 (4; KO); ventral TA-CA1, *n* = 9 (4; WT), *n* = 10 (4; KO), ns, not significant, two-way repeated-measures ANOVA with Holm-Sidak multiple comparison test (**A**: interaction, *F*_(11,187)_ = 0.1489, *p* = 0.9994; genotype, *F*_(1,17)_ = 0.0017, *p* = 0.9675; fiber volley, *F*_(11,187)_ = 423.8, *p* < 0.0001; **B**: interaction, *F*_(6,84)_ = 0.1474, *p* = 0.9891; fiber volley, *F*_(6,84)_ = 180.8, *p* < 0.0001; genotype, *F*_(1,14)_ = 0.1284, *p* = 0.7254; **C**: interaction, *F*_(9,198)_ = 1.097, *p* = 0.3663; fiber volley, *F*_(9,198)_ = 266.7, *p* < 0.0001; genotype, *F*_(1,22)_ = 1.564, *p* = 0.2242; **D**: interaction, *F*_(6,102)_ = 0.1219, *p* = 0.9935; fiber volley, *F*_(6,102)_ = 471.5, *p* < 0.0001; genotype, *F*_(1,17)_ = 0.09676, *p* = 0.7595). **(E–H)** Normal paired-pulse facilitation in the ventral SC-CA1 pathway **(E)**, ventral TA-CA1 pathway **(F)**, dorsal SC-CA1 pathway **(G)**, and dorsal TA-CA1 pathway **(H)** in the hippocampal CA1 region of *Lrrc4c*^−/−^ mice (2–4 months; male). Dorsal SC-CA1, *n* = 9 slices from three mice (WT), *n* = 10 (3; KO); dorsal TA-CA1, *n* = 8 (3; WT), *n* = 8 (3; KO); ventral SC-CA1, *n* = 12 (4; WT), *n* = 12 (4; KO); ventral TA-CA1, *n* = 9 (4; WT), *n* = 10 (4; KO), ns, not significant, two-way repeated-measures ANOVA with Holm-Sidak multiple comparison test (**E**: interaction, *F*_(5,80)_ = 0.6057, *p* = 0.6957; interval, *F*_(5,80)_ = 59.11, *p* < 0.0001; genotype, *F*_(1,16)_ = 2.371, *p* = 0.1432; **F**: interaction, *F*_(5,75)_ = 1.058, *p* = 0.3903; interval, *F*_(5,75)_ = 151.3, *p* < 0.0001; genotype, *F*_(1,15)_ = 0.5615, *p* = 0.4653; **G**: interaction, *F*_(5,105)_ = 1.185, *p* = 0.3213; interval, *F*_(5,105)_ = 269.5, *p* < 0.0001; genotype, *F*_(1,21)_ = 0.7974, *p* = 0.3820; **H**: interaction, *F*_(5,80)_ = 1.02, *p* = 0.4118; interval, *F*_(5,80)_ = 72.76, *p* < 0.0001; genotype, *F*_(1,16)_ = 0.02833, *p* = 0.8684).

However, these whole-cell recordings might fail to detect dendritic layer-specific changes in synaptic transmission, such as those occurring at NGL-1–enriched TA-CA1 synapses in the SLM layer, which might dissipate while traveling the distance from the SLM layer to the cell body or could experience interference from unaffected inputs at SC-CA1 synapses in the SR layer. However, both *Lrrc4c^−/−^* TA-CA1 and SC-CA1 pathways displayed normal basal synaptic transmission in ventral and dorsal CA1 regions (Figures 4A–D). Furthermore, paired-pulse facilitation was unaffected in these pathways (Figures 4E–H), suggestive of normal presynaptic release. These results collectively suggest that *Lrrc4c* deletion does not affect spontaneous synaptic transmission in CA1 neurons or evoked transmission in hippocampal TA-CA1 and SC-CA1 pathways.

### Suppression of Short-Term Synaptic Plasticity in *Lrrc4c*^−/−^ TA-CA1 and SC-CA1 Pathways

**Figure 5:**
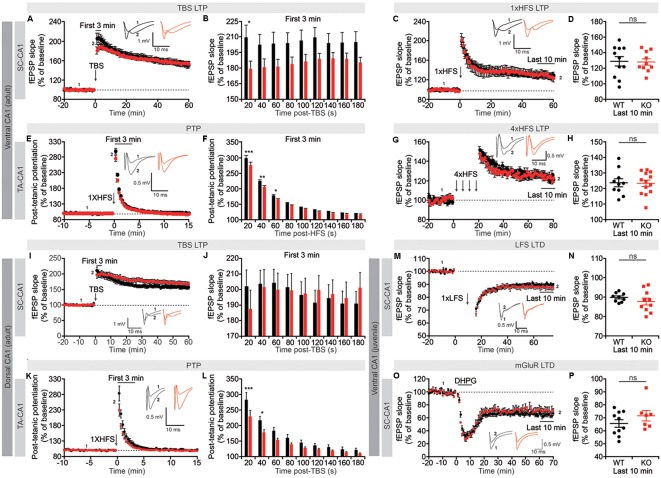
Suppression of short-term synaptic plasticity, but normal long-term potentiation (LTP) and long-term depression (LTD), in *Lrrc4c^−/−^* TA-CA1 and SC-CA1 pathways. **(A,B)** Normal LTP induced by theta-burst stimulation (TBS), but suppressed short-term potentiation, in the ventral SC-CA1 pathway in *Lrrc4c*^−/−^ mice (2–4 months; male). Note that short-term potentiation is significantly decreased during the first 20 s after stimulation of the ventral SC-CA1 pathway. *n* = 17 slices from four mice (WT), *n* = 19 (5; KO), *p < 0.05, two-way repeated-measures ANOVA with Holm-Sidak multiple comparison test (**B**: interaction, *F*_(8,272)_ = 2.437, *p* = 0.0147; time, *F*_(8,272)_ = 2.572, *p* = 0.0101; genotype, *F*_(1,34)_ = 3.155, *p* = 0.0846; 20 s, *p* = 0.0152). **(C,D)** Normal LTP induced by high-frequency stimulation (HFS) of the ventral SC-CA1 pathway in *Lrrc4c*^−/−^ mice (2–4 months; male). *n* = 11 (4; WT), *n* = 10 (4; KO), ns, not significant, Student’s *t*-test (**D**: *t*_(19)_ = 0.1175; *p* = 0.9077). **(E,F)** Suppressed post-tetanic potentiation (PTP) induced by 1× HFS of the ventral TA-CA1 pathway in *Lrrc4c*^−/−^ mice (2–4 months; male). Note the decreased PTP during the first 60 s after stimulation of the ventral TA-CA1 pathway. *n* = 10 (3; WT), *n* = 11 (3; KO), *p < 0.05, **p < 0.01, ***p < 0.001, ns, not significant, two-way repeated-measures ANOVA with Holm-Sidak multiple comparison test (**F**: interaction, *F*_(8,152)_ = 2.4, *p* = 0.0182; time, *F*_(8,152)_ = 2.572, *p* < 0.0001; genotype, *F*_(1,19)_ = 6.6321, *p* = 0.0185; 20 s, *p* < 0.0001, 40 s, *p* = 0.0014, 60 s, *p* = 0.0479). **(G,H)** Normal LTP induced by 4× HFS of the ventral TA-CA1 pathway in *Lrrc4c*^−/−^ mice (2–4 months; male). *n* = 10 (3; WT), *n* = 13 (3; KO), ns, not significant, Student’s *t*-test (**H**: Student’s *t*-test; *t*_(21)_ = 0.1278; *p* = 0.8995). **(I,J)** Normal LTP induced by TBS and normal short-term potentiation in the dorsal SC-CA1 pathway in *Lrrc4c*^−/−^ mice (2–4 months; male). *n* = 11 (5; WT), *n* = 16 (5; KO), two-way repeated-measures ANOVA with Holm-Sidak multiple comparison test (**J**: interaction, *F*_(8,192)_ = 2.993, *p* = 0.0035; time, *F*_(8,192)_ = 2.662, *p* = 0.0086; genotype, *F*_(1, 24)_ = 0.001075, *p* = 0.9741). **(K,L)** Suppressed PTP induced by 1× HFS of the dorsal TA-CA1 pathway in *Lrrc4c*^−/−^ mice (2–4 months; male). Note the decreased PTP during the first 40 s after stimulation of the ventral TA-CA1 pathway. *n* = 8 (4; WT), *n* = 11 (4; KO), *p < 0.05, ***p < 0.001, two-way repeated-measures ANOVA with Holm-Sidak multiple comparison test (**L**: interaction, *F*_(8,136)_ = 2.891, *p* = 0.0053; time, *F*_(8,136)_ = 283, *p* < 0.0001; genotype, *F*_(1,17)_ = 6.033, *p* = 0.0251; 20 s, *p* = 0.0005, 40 s, *p* = 0.0222). **(M,N)** Normal LTD induced by low-frequency stimulation (LFS; 1 Hz, 900 pulses) of the ventral SC-CA1 pathway in *Lrrc4c*^−/−^ mice (3 weeks; male). *n* = 9 (4; WT), *n* = 10 (4; KO), ns, not significant, Student’s *t*-test (**N**: *t*_(17)_ = 1.062; *p* = 0.3033). **(O,P)** Normal LTD induced by activation of metabotropic glutamate receptors with dihydroxyphenylglycine (DHPG; 50 μM for 5 min) in the ventral SC-CA1 pathway in *Lrrc4c*^−/−^ mice (3 weeks; male). *n* = 10 (4; WT), *n* = 7 (3; KO), ns, not significant, Student’s *t*-test (**P**: *t*_(15)_ = 1.236; *p* = 0.2356).

Although spontaneous and basal transmissions were not affected in the *Lrrc4c^−/−^* hippocampus, *Lrrc4c* deletion might affect synaptic plasticity. We thus next tested changes in synaptic plasticity by measuring LTP induced by TBS or HFS. The *Lrrc4c*^−/−^ hippocampus displayed normal TBS-LTP and HFS-LTP in the SC-CA1 pathway in the ventral CA1 region (Figures 5A–D). fEPSP responses during the first 20 s immediately after application of TBS, referred to hereafter as short-term potentiation, was significantly decreased; subsequent recordings also displayed a similar decreasing tendency, although these changes did not reach statistical significance (Figures 5A,B).

Unlike the SC-CA1 pathway, the TA-CA1 pathway in the SLM layer was highly resistant to LTP induction, with a single application of HFS failing to produce long-lasting changes (Figure 5E). However, this single HFS did reveal a significant decrease in early fEPSP responses during the first 60 s post-stimulus, a phenomenon known as PTP that involves largely presynaptic changes (Zucker and Regehr, 2002), similar to the suppressed short-term potentiation observed in TBS-LTP in the SC-CA1 pathway (Figures 5E,F). Induction of LTP in the ventral TA-CA1 pathway by applying HFS four times separated by 5-min intervals revealed no differences between genotype (Figures 5G,H), a finding similar to the normal TBS-LTP and HFS-LTP in the SC-CA1 pathway.

In the dorsal CA1 region, neither TBS-LTP nor short-term potentiation immediately after TBS delivery was affected in the SC-CA1 pathway, whereas PTP was suppressed in the TA-CA1 pathway, results partially similar to those obtained in the ventral CA1 region (Figures 5I–L). Lastly, neither long-term depression (LTD) induced by low-frequency stimulation (LFS) nor dihydroxyphenylglycine (DHPG) treatment (for mGluR-LTD) was affected in the SC-CA1 pathway in the ventral CA1 region of *Lrrc4c^−/−^* mice (Figures 5M–P).

These results collectively suggest that NGL-1 is important for short-term synaptic plasticity (short-term potentiation and PTP) in both SC-CA1 and TA-CA1 pathways in the *Lrrc4c^−/−^* ventral hippocampus, but not for long-term plasticity [LTP (TBS and HFS) or LTD (LFS and mGluR)]. Moreover, given that NGL-1 is enriched in the TA-CA1 pathway in the SLM layer, changes in short-term plasticity in the SC-CA1 pathway in addition to the TA-CA1 pathway in the ventral hippocampus suggest both input-specific and input-nonspecific impairments, although the dorsal hippocampus seems to display an input-specific impairment in PTP in the TA-CA1 pathway but not short-term plasticity in the SC-CA1 pathway.

**Figure 6:**
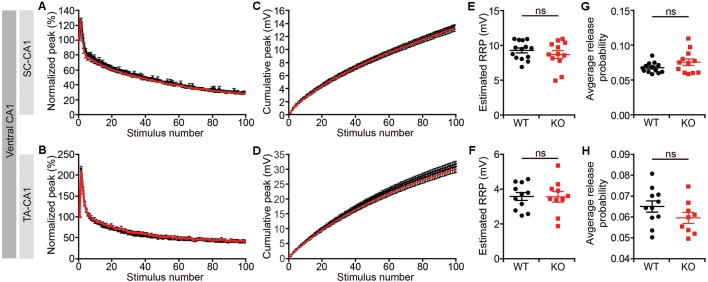
Normal readily releasable pool and release probability *Lrrc4c^−/−^* TA-CA1 and SC-CA1 pathways. **(A,B,E,F)** Normalized **(A,E)** and cumulative **(B,F)** field EPSP (fEPSP) responses at hippocampal SC-CA1 **(A,B)** and TA-CA1 **(E,F)** synapses (2–4 months; male) to repetitive stimulations (20 Hz, 5 s). **(C,G)** Normal readily releasable pool at *Lrrc4c*^−/−^ SC-CA1 **(C)** and TA-CA1 **(G)** synapses. *n* = 14 slices from four mice (WT), *n* = 12 (4; KO) for SR, *n* = 11 (4; WT), *n* = 10 (4; KO) for SLM, ns, not significant, Student’s *t*-test (**E**: *t*_(24)_ = 0.9234; *p* = 0.3650; **F**: *t*_(19)_ = 0.0520; *p* = 0.3650). **(D,H)** Normal release probability (p_r_) at *Lrrc4c*^−/−^ SC-CA1 **(D)** and TA-CA1 **(H)** synapses. The release probability was measured by dividing the first fEPSP amplitude by the pool size estimate. *n* = 14 slices from four mice (WT), *n* = 12 (4; KO) for SR, *n* = 11 (4; WT), *n* = 10 (4; KO) for SLM, ns, not significant, Mann-Whitney test **(G)** and Student’s *t*-test (**H**; **G**: *U* = 57; *p* = 0.2701; **H**: *t*_(18)_ = 1.431; *p* = 0.1695).

Given that short-term synaptic plasticity mainly involves presynaptic changes (Zucker and Regehr, 2002), we measured readily releasable pool estimates and average release probabilities in the ventral CA1, as described previously (Han et al., 2011). We found no changes in either the size of the readily releasable pool or release probabilities for both the *Lrrc4c^−/−^* SR and SLM layers (Figures 6A–H). These results suggest that *Lrrc4c* deletion induces impaired short term plasticity through mechanisms independent of the readily releasable pool and release probability.

### Altered Synaptic Protein Localization in SR and SLM Layers of *Lrrc4c*^−/−^ CA1 Neurons

**Figure 7:**
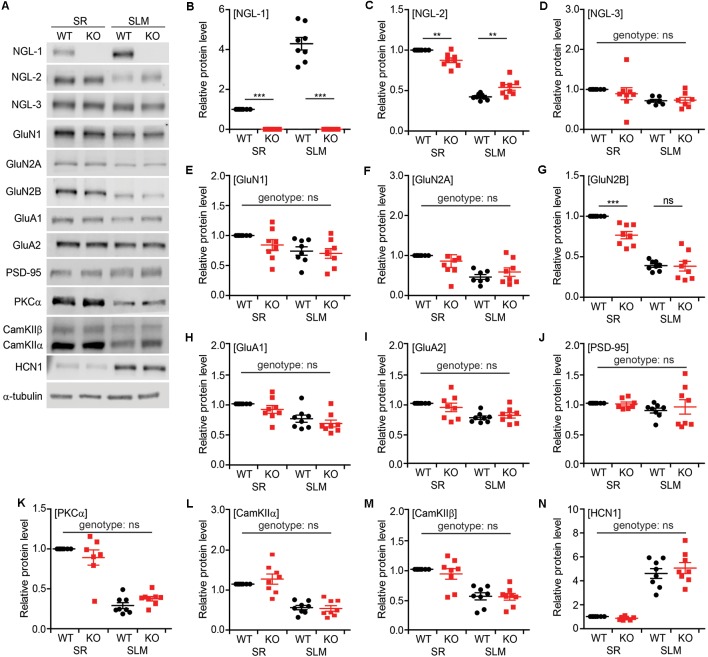
Altered synaptic protein localization in SR and SLM layers in *Lrrc4c^−/−^* CA1 neurons. **(A)** Representative images of immunoblots for synaptic proteins in SR and SLM layers of the ventral hippocampus from WT and *Lrrc4c*^−/−^ mice (3 months; male). **(B–N)** Relative levels of synaptic proteins in SR and SLM layers, normalized to WT-SR levels. Note that levels of NGL-2 and GluN2B are decreased in the SR region of *Lrrc4c*^−/−^ mice. Immunoblotted proteins include NGL family proteins **(B–D)**, N-methyl-D-aspartate receptor (NMDAR) subunits **(E–G)**, α-amino-3-hydroxy-5-methyl-4-isoxazolepropionic acid receptor (AMPAR) subunits **(H,I)**, PSD-95 (**J**; postsynaptic scaffold), signaling molecules **(K–M)**, and HCN1 (**N**; SLM-enriched). *n* = 8 mice (WT), *n* = 8 (KO), **p < 0.01, ***p < 0.001, ns, not significant, two-way ANOVA with Holm-Sidak multiple comparison test (**B**: interaction, *F*_(1,28)_ = 109.7, *p* < 0.001; genotype, *F*_(1,28)_ = 283.4, *p* < 0.001; layer, *F*_(1,28)_ = 109.7, *p* < 0.001; SR, *p* < 0.001, SLM, *p* < 0.001; **C**: interaction, *F*_(1,28)_ = 26.02, *p* < 0.001; genotype, *F*_(1,28)_ = 0.03946, *p* = 0.8440; layer, *F*_(1,28)_ = 371.4, *p* < 0.00; SR, *p* = 0.0016, SLM, *p* = 0.0017; **D**: interaction, *F*_(1,26)_ = 0.4848, *p* = 0.4924; genotype, *F*_(1,26)_ = 0.25, *p* = 0.6213; layer, *F*_(1,26)_ = 6.242, *p* = 0.0191; **E**: interaction, *F*_(1,28)_ = 0.7101, *p* = 0.4066; genotype, *F*_(1,28)_ = 1.988, *p* = 0.1695; layer, *F*_(1,28)_ = 8.217, *p* = 0.0078; **F**: interaction, *F*_(1,27)_ = 1.619, *p* = 0.2141; genotype, *F*_(1,27)_ = 0.005529, *p* = 0.9413; layer, *F*_(1,27)_ = 15.41, *p* = 0.0005; **G**: interaction, *F*_(1,28)_ = 8.519, *p* = 0.0069; genotype, *F*_(1,28)_ = 9.523, *p* = 0.0045; layer, *F*_(1,28)_ = 163.6, *p* < 0.001; SR, *p* = 0.0004, SLM, *p* = 0.9067; **H**: interaction, *F*_(1,28)_ = 0.01422, *p* = 0.9059; genotype, *F*_(1,28)_ = 2.666, *p* = 0.1137; layer, *F*_(1,28)_ = 21.16, *p* < 0.0001; **I**: interaction, *F*_(1,28)_ = 1.481, *p* = 0.2337; genotype, *F*_(1,28)_ = 0.1069, *p* = 0.7461; layer, *F*_(1,28)_ = 17.92, *p* = 0.0002; **J**: interaction, *F*_(1,28)_ = 0.3065, *p* = 0.5842; genotype, *F*_(1,28)_ = 1.092, *p* = 0.3049; layer, *F*_(1,28)_ = 2.359, *p* = 0.1358; **K**: interaction, *F*_(1,27)_ = 3.882, *p* = 0.0591; genotype, *F*_(1,27)_ = 0.04942, *p* = 0.8258; layer, *F*_(1,27)_ = 155, *p* < 0.001; **L**: interaction, *F*_(1,28)_ = 0.9144, *p* = 0.3471; genotype, *F*_(1,28)_ = 0.4595, *p* = 0.5034; layer, *F*_(1,28)_ = 73.57, *p* < 0.001; **M**: interaction, *F*_(1,28)_ = 0.3264, *p* = 0.5723; genotype, *F*_(1,28)_ = 0.4964, *p* = 0.4869; layer, *F*_(1,28)_ = 48.02, *p* < 0.001; **N**: interaction, *F*_(1,28)_ = 0.9428, *p* = 0.3399; genotype, *F*_(1,28)_ = 0.2563, *p* = 0.6167; layer, *F*_(1,28)_ = 159, *p* < 0.001).

To examine biochemical changes associated with the decreased PSD density and short-term synaptic plasticity observed in SR and SLM layers of the *Lrrc4c^−/−^* hippocampus, we dissected SR- and SLM-enriched lysates from ventral hippocampal CA1 regions and measured the levels of various synaptic proteins by immunoblotting (Figure 7A). These samples were validated using NGL-1 (SLM-enriched), NGL-2 (SR-enriched), GluN2B (SR-enriched; Nicholson et al., 2006), and HCN1 (SLM-enriched; Nolan et al., 2004) as positive controls (see below for details Figures 7B,C,G,N).

The NGL family proteins NGL-1 and NGL-2 showed SLM- and SR-specific distribution patterns, respectively, as previously reported (Nishimura-Akiyoshi et al., 2007), whereas NGL-3 was similarly distributed to both layers (Figures 7B–D), suggesting that NGL-3 may function in both TA-CA1 and SC-CA1 pathways. Intriguingly, NGL-2 was significantly decreased in the SR layer and increased in the SLM layer of *Lrrc4c^−/−^* CA1 neurons, suggesting translocation of NGL-2 from the SR layer to the SLM layer in the absence of NGL-1 in the SLM layer (Figure 7B).

Because NGL-2 is known to biochemically associate with N-methyl-D-aspartate receptors (NMDARs; Kim et al., 2006; Um et al., 2018), we also examined the NMDAR subunits, GluN1, GluN2A, and GluN2B. Interestingly, GluN2B levels were significantly decreased in the SR, but not SLM, layer of the *Lrrc4c^−/−^* hippocampus, whereas GluN1 or GluN2A levels were not affected (Figures 7E–G).

We also tested the α-amino-3-hydroxy-5-methyl-4-isoxazolepropionic acid (AMPA) receptor subunits, GluA1 and GluA2, and PSD-95 (excitatory postsynaptic scaffold), but found no differences between genotypes (Figures 7H–J). Similarly, PKCα, calcium-dependent kinase IIα (CamKIIα) and CamKIIβ—signaling molecules associated with short-term plasticity—were normal in the *Lrrc4c^−/−^* hippocampus (Figures 7K–M).

Collectively, these data indicate that deletion of *Lrrc4c*, which is enriched in the SLM layer, leads to the translocation of NGL-2 from the SR layer to the SLM layer, as well as a decrease in SR-specific localization of GluN2B in ventral CA1 neurons.

## Discussion

In the present study, we explored the impact of *Lrrc4c* deletion on excitatory synapse development and function in mice. Overall, our results indicate that *Lrrc4c* deletion leads to moderate changes in excitatory synapse development and function in the hippocampus.

Our EM results indicate that NGL-1 deletion leads to moderate decreases in PSD density in SR and SLM layers of the ventral, but not dorsal, CA1 region of *Lrrc4c^−/−^* mice (Figures 2C,D). This result is interesting in that: (1) only the ventral region is affected; and (2) both SR and SLM layers are affected, despite the fact that NGL-1 is ~4-times more strongly expressed in the SLM layer (Figure 7B). The ventral-only decrease in PSD density in the *Lrrc4c*^−/−^ hippocampus will require further investigation, although dorsal and ventral regions of the hippocampus are known to have different properties and serve different brain functions; i.e., the dorsal hippocampus subserves for cognitive functions, while the ventral hippocampus subserves for emotion-related functions such as anxiety and stress (Fanselow and Dong, 2010). However, both dorsal and ventral hippocampus receive presynaptic inputs originating from the EC; more specifically, medial and lateral EC layer III neurons project to the proximal (closer to CA2) and distal (closer to the subiculum) CA1 regions, respectively, in both dorsal and ventral hippocampus (Canto et al., 2008; Heys et al., 2012; Masurkar et al., 2017). Therefore, the ventral-specific decrease in PSD density in the *Lrrc4c*^−/−^ SLM layer suggests a stronger impact of NGL-1 deletion on ventral TA-CA1 synapses originating from more ventral and medial EC layer III, although functional changes at TA-CA1 synapses were observed in both ventral and dorsal hippocampus in *Lrrc4c*^−/−^ mice (see below).

The decreased PSD density in the SLM layer induced by the loss of NGL-1 may be attributable to the loss of the trans-synaptic interaction between postsynaptic NGL-1 and presynaptic netrin-G1 in the axons of SLM-projecting EC layer III neurons (Niimi et al., 2007; Nishimura-Akiyoshi et al., 2007; Matsukawa et al., 2014), a neuronal population that differs from dentate gyrus-projecting neurons in the EC layer II (Canto et al., 2008; Heys et al., 2012; Masurkar et al., 2017). The decreased PSD density in the SR layer induced by the loss of SLM-enriched NGL-1 might be attributable to the partial shift of NGL-2 localization from the SR to the SLM layer (Figure 7C). The NGL-2 protein translocated to the SLM layer might be stabilized through a non-conventional NGL-2–netrin-G1 interaction with a much weaker affinity relative to that for the conventional NGL-1–netrin-G1 interaction (Woo et al., 2009b; Seiradake et al., 2011), enabled by the absence of NGL-1 at SLM synapses. Alternatively, the decrease in SR levels of GluN2B (Figure 7G), known to form a complex with NGL-2 (Kim et al., 2006; Um et al., 2018; but see Zhang et al., 2016), might have contributed to the decreased PSD density in the SR layer. These layer-independent impacts of *Lrrc4c* deletion on both SR and SLM layers contrast with the input-specific decrease in spine density and excitatory synaptic transmission in the SR layer induced by *Lrrc4* (NGL-2) deletion in mice (DeNardo et al., 2012; Um et al., 2018) and suggest the critical roles of SLM synapses for hippocampal functions. Previous studies have shown that direct input from the EC layer III to the CA1 SLM layer is important for many hippocampal functions (Remondes and Schuman, 2002, 2003, 2004; Brun et al., 2008; Suh et al., 2011). Therefore, the SR-to-SLM translocation of NGL-2 in *Lrrc4c*^−/−^ mice may represent an effort by CA1 neurons to mitigate the impacts of *Lrrc4c* (NGL-1) deletion on the TA-CA1 pathway.

*Lrrc4c^−/−^* mice show normal levels of spontaneous and basal excitatory synaptic transmission in the ventral hippocampus (Figures 3A,C, 4A,B,E,F). These results contrast with the moderately decreased density of the PSD in this region. One possible explanation for this discrepancy is that the SLM layer is located in the distal (not proximal) segment of CA1 neuronal dendrites, dampening the impact of the reduced density of the PSD on spontaneous synaptic transmission (i.e., mEPSCs) measured in cell bodies. Alternatively, it could be that the moderate reduction in the density of the PSD (~10%–15%) is not sufficient to manifest as functional changes (i.e., basal transmission). With regards to amplitude, it could be that only synapses unaffected by *Lrrc4c* deletion were left, thus leaving the average amplitude unchanged. Lastly, there is the possibility of compensatory pre- or postsynaptic changes. Again, the NGL-2 protein translocated from the SR to SLM layer might dampen the functional impairments at TA-CA1 synapses.

Intriguingly, however, short-term synaptic plasticity (short-term potentiation and PTP) was suppressed in SC-CA1 and TA-CA1 pathways in the ventral *Lrrc4c^−/−^* hippocampus, while only the PTP in the TA-CA1 pathway, but not short-term potentiation in the SC-CA1 pathway, was suppressed in the dorsal *Lrrc4c*^−/−^ hippocampus (Figure 5). These results indicate stronger and input-specific impacts of *Lrrc4c* deletion in the TA-CA1 pathway relative to the SC-CA1 pathway. Given that TA-CA1 synapses are the place where postsynaptic NGL-1 trans-synaptically interacts with presynaptic netrin-G1 (Niimi et al., 2007; Nishimura-Akiyoshi et al., 2007) and that short-term potentiation and PTP are known to involve presynaptic mechanisms (Zucker and Regehr, 2002), the suppressed PTP at *Lrrc4c*^−/−^ TA-CA1 synapses might involve limited presynaptic netrin-G1 localization at *Lrrc4c*^−/−^ synapses, as recently reported (Matsukawa et al., 2014). Alternatively, but not mutually exclusively, it may involve limited cis-interaction of presynaptic netrin-G1 with neighboring LAR-family receptor tyrosine phosphatases, an interaction induced by the trans-synaptic binding of NGL-1 to netrin-G1 that promotes presynaptic differentiation (Song et al., 2013). Similarly, the suppressed short-term plasticity at *Lrrc4c*^−/−^ SC-CA1 synapses in the SR layer may reflect decreased levels of presynaptic netrin-G2 caused by the reduction in postsynaptic NGL-2 levels, which in turn is attributable to the SR-to-SLM shift in NGL-2 protein.

NGL-1 has also been implicated in several neuropsychiatric disorders. A single-nucleotide polymorphism in the *LRRC4C* gene is associated with the risk of bipolar disorder (Greenwood et al., 2012). In addition, copy number variations in *LRRC4C* have been identified in individuals with autism spectrum disorders (ASDs) and developmental delays (Maussion et al., 2017). Some of the synaptic phenotypes that are associated with *Lrrc4c* deletion identified in the present study might underlie these NGL-1-related brain disorders.

In conclusion, our results suggest that *Lrrc4c* deletion in mice leads to input-independent changes in the development and function at excitatory synapses in the SLM and SR layers of the ventral hippocampus.

## Data Availability

This manuscript contains previously unpublished data. The name of the repository and accession number are not available.

## Ethics Statement

Mouse maintenance and procedures were performed in accordance with the Requirements of Animal Research at KAIST, and all procedures were approved by the Committee of Animal Research at KAIST (KA2012-19).

## Author Contributions

YiC, HP, HK, SeyeongK, SL and WS performed electrophysiological experiments. YiC, SeyeonK and JK performed Lac Z staining experiment. YiC, HP and HJ performed immunoblot experiments. HH, YeC and YB performed electron microscopy experiment. EK designed research and wrote the manuscript.

## Conflict of Interest Statement

The authors declare that the research was conducted in the absence of any commercial or financial relationships that could be construed as a potential conflict of interest.

## References

[B1] Bemben M. A. Shipman S. L. Nicoll R. A. Roche K. W. (2015). The cellular and molecular landscape of neuroligins. Trends Neurosci. 38, 496–505. 10.1016/j.tins.2015.06.004 26209464 PMC9381026

[B2] Brun V. H. Leutgeb S. Wu H. Q. Schwarcz R. Witter M. P. Moser E. I.. (2008). Impaired spatial representation in CA1 after lesion of direct input from entorhinal cortex. Neuron 57, 290–302. 10.1016/j.neuron.2007.11.034 18215625

[B3] Canto C. B. Wouterlood F. G. Witter M. P. (2008). What does the anatomical organization of the entorhinal cortex tell us? Neural Plast. 2008:381243. 10.1155/2008/381243 18769556 PMC2526269

[B5] DeNardo L. A. de Wit J. Otto-Hitt S. Ghosh A. (2012). NGL-2 regulates input-specific synapse development in CA1 pyramidal neurons. Neuron 76, 762–775. 10.1016/j.neuron.2012.10.013 23177961 PMC7566585

[B4] de Wit J. Ghosh A. (2016). Specification of synaptic connectivity by cell surface interactions. Nat. Rev. Neurosci. 17, 22–35. 10.1038/nrn.2015.3 26656254

[B6] Fanselow M. S. Dong H. W. (2010). Are the dorsal and ventral hippocampus functionally distinct structures? Neuron 65, 7–19. 10.1016/j.neuron.2009.11.031 20152109 PMC2822727

[B7] Greenwood T. A. Akiskal H. S. Akiskal K. K. Bipolar Genome Study Kelsoe J. R. (2012). Genome-wide association study of temperament in bipolar disorder reveals significant associations with three novel Loci. Biol. Psychiatry 72, 303–310. 10.1016/j.biopsych.2012.01.018 22365631 PMC3925336

[B8] Han Y. Kaeser P. S. Sudhof T. C. Schneggenburger R. (2011). RIM determines Ca^2+^ channel density and vesicle docking at the presynaptic active zone. Neuron 69, 304–316. 10.1016/j.neuron.2010.12.014 21262468 PMC3259453

[B9] Heys J. G. Schultheiss N. W. Shay C. F. Tsuno Y. Hasselmo M. E. (2012). Effects of acetylcholine on neuronal properties in entorhinal cortex. Front. Behav. Neurosci. 6:32. 10.3389/fnbeh.2012.00032 22837741 PMC3402879

[B11] Kim S. Burette A. Chung H. S. Kwon S. K. Woo J. Lee H. W.. (2006). NGL family PSD-95-interacting adhesion molecules regulate excitatory synapse formation. Nat. Neurosci. 9, 1294–1301. 10.1038/nn1763 16980967

[B10] Kim M. H. Choi J. Yang J. Chung W. Kim J. H. Paik S. K.. (2009). Enhanced NMDA receptor-mediated synaptic transmission, enhanced long-term potentiation and impaired learning and memory in mice lacking IRSp53. J. Neurosci. 29, 1586–1595. 10.1523/JNEUROSCI.4306-08.2009 19193906 PMC6666077

[B12] Ko J. Choii G. Um J. W. (2015). The balancing act of GABAergic synapse organizers. Trends Mol. Med. 21, 256–268. 10.1016/j.molmed.2015.01.004 25824541

[B13] Krueger D. D. Tuffy L. P. Papadopoulos T. Brose N. (2012). The role of neurexins and neuroligins in the formation, maturation, and function of vertebrate synapses. Curr. Opin. Neurobiol. 22, 412–422. 10.1016/j.conb.2012.02.012 22424845

[B14] Kwon S. K. Woo J. Kim S. Y. Kim H. Kim E. (2010). Trans-synaptic adhesions between netrin-G ligand-3 (NGL-3) and receptor tyrosine phosphatases LAR, protein-tyrosine phosphatase delta (PTPdelta), and PTPsigma via specific domains regulate excitatory synapse formation. J. Biol. Chem. 285, 13966–13978. 10.1074/jbc.m109.061127 20139422 PMC2859559

[B15] Lee H. Lee E. J. Song Y. S. Kim E. (2014). Long-term depression-inducing stimuli promote cleavage of the synaptic adhesion molecule NGL-3 through NMDA receptors, matrix metalloproteinases and presenilin/γ-secretase. Philos. Trans. R. Soc. Lond. B Biol. Sci. 369:20130158. 10.1098/rstb.2013.0158 24298159 PMC3843889

[B16] Lin J. C. Ho W. H. Gurney A. Rosenthal A. (2003). The netrin-G1 ligand NGL-1 promotes the outgrowth of thalamocortical axons. Nat. Neurosci. 6, 1270–1276. 10.1038/nn1148 14595443

[B17] Masurkar A. V. Srinivas K. V. Brann D. H. Warren R. Lowes D. C. Siegelbaum S. A. (2017). Medial and lateral entorhinal cortex differentially excite deep versus superficial CA1 pyramidal neurons. Cell Rep. 18, 148–160. 10.1016/j.celrep.2016.12.012 28052245 PMC5381513

[B18] Matsukawa H. Akiyoshi-Nishimura S. Zhang Q. Luján R. Yamaguchi K. Goto H.. (2014). Netrin-G/NGL complexes encode functional synaptic diversification. J. Neurosci. 34, 15779–15792. 10.1523/JNEUROSCI.1141-14.2014 25411505 PMC6608433

[B19] Maussion G. Cruceanu C. Rosenfeld J. A. Bell S. C. Jollant F. Szatkiewicz J.. (2017). Implication of LRRC4C and DPP6 in neurodevelopmental disorders. Am. J. Med. Genet. A 173, 395–406. 10.1002/ajmg.a.38021 27759917 PMC5833302

[B20] Missler M. Südhof T. C. Biederer T. (2012). Synaptic cell adhesion. Cold Spring Harb. Perspect. Biol. 4:a005694. 10.1101/cshperspect.a005694 22278667 PMC3312681

[B21] Nakashiba T. Ikeda T. Nishimura S. Tashiro K. Honjo T. Culotti J. G.. (2000). Netrin-G1: a novel glycosyl phosphatidylinositol-linked mammalian netrin that is functionally divergent from classical netrins. J. Neurosci. 20, 6540–6550. 10.1523/JNEUROSCI.20-17-06540.2000 10964959 PMC6772945

[B22] Nakashiba T. Nishimura S. Ikeda T. Itohara S. (2002). Complementary expression and neurite outgrowth activity of netrin-G subfamily members. Mech. Dev. 111, 47–60. 10.1016/s0925-4773(01)00600-1 11804778

[B23] Nicholson D. A. Trana R. Katz Y. Kath W. L. Spruston N. Geinisman Y. (2006). Distance-dependent differences in synapse number and AMPA receptor expression in hippocampal CA1 pyramidal neurons. Neuron 50, 431–442. 10.1016/j.neuron.2006.03.022 16675397

[B24] Niimi K. Nishimura-Akiyoshi S. Nakashiba T. Itohara S. (2007). Monoclonal antibodies discriminating netrin-G1 and netrin-G2 neuronal pathways. J. Neuroimmunol. 192, 99–104. 10.1016/j.jneuroim.2007.09.026 17945353

[B25] Nishimura-Akiyoshi S. Niimi K. Nakashiba T. Itohara S. (2007). Axonal netrin-Gs transneuronally determine lamina-specific subdendritic segments. Proc. Natl. Acad. Sci. U S A 104, 14801–14806. 10.1073/pnas.0706919104 17785411 PMC1964543

[B26] Nolan M. F. Malleret G. Dudman J. T. Buhl D. L. Santoro B. Gibbs E.. (2004). A behavioral role for dendritic integration: HCN1 channels constrain spatial memory and plasticity at inputs to distal dendrites of CA1 pyramidal neurons. Cell 119, 719–732. 10.1016/j.cell.2004.11.020 15550252

[B27] Remondes M. Schuman E. M. (2002). Direct cortical input modulates plasticity and spiking in CA1 pyramidal neurons. Nature 416, 736–740. 10.1038/416736a 11961555

[B28] Remondes M. Schuman E. M. (2003). Molecular mechanisms contributing to long-lasting synaptic plasticity at the temporoammonic-CA1 synapse. Learn. Mem. 10, 247–252. 10.1101/lm.59103 12888542 PMC202314

[B29] Remondes M. Schuman E. M. (2004). Role for a cortical input to hippocampal area CA1 in the consolidation of a long-term memory. Nature 431, 699–703. 10.1038/nature02965 15470431

[B30] Seiradake E. Coles C. H. Perestenko P. V. Harlos K. McIlhinney R. A. Aricescu A. R.. (2011). Structural basis for cell surface patterning through NetrinG-NGL interactions. EMBO J. 30, 4479–4488. 10.1038/emboj.2011.346 21946559 PMC3230378

[B31] Shen K. Scheiffele P. (2010). Genetics and cell biology of building specific synapse connectivity. Annu. Rev. Neurosci. 33, 473–507. 10.1146/annurev.neuro.051508.135302 20367446 PMC3082953

[B32] Sheng M. Hoogenraad C. C. (2007). The postsynaptic architecture of excitatory synapses: a more quantitative view. Annu. Rev. Biochem. 76, 823–847. 10.1146/annurev.biochem.76.060805.160029 17243894

[B33] Sheng M. Kim E. (2011). The postsynaptic organization of synapses. Cold Spring Harb. Perspect. Biol. 3:a005678. 10.1101/cshperspect.a005678 22046028 PMC3225953

[B34] Sheng M. Sala C. (2001). PDZ domains and the organization of supramolecular complexes. Annu. Rev. Neurosci. 24, 1–29. 10.1146/annurev.neuro.24.1.1 11283303

[B35] Song Y. S. Lee H. J. Prosselkov P. Itohara S. Kim E. (2013). Trans-induced cis interaction in the tripartite NGL-1, netrin-G1 and LAR adhesion complex promotes development of excitatory synapses. J. Cell Sci. 126, 4926–4938. 10.1242/jcs.129718 23986473

[B36] Sudhof T. C. (2008). Neuroligins and neurexins link synaptic function to cognitive disease. Nature 455, 903–911. 10.1038/nature07456 18923512 PMC2673233

[B37] Sudhof T. C. (2017). Synaptic neurexin complexes: a molecular code for the logic of neural circuits. Cell 171, 745–769. 10.1016/j.cell.2017.10.024 29100073 PMC5694349

[B38] Sudhof T. C. (2018). Towards an understanding of synapse formation. Neuron 100, 276–293. 10.1016/j.neuron.2018.09.040 30359597 PMC6226307

[B39] Suh J. Rivest A. J. Nakashiba T. Tominaga T. Tonegawa S. (2011). Entorhinal cortex layer III input to the hippocampus is crucial for temporal association memory. Science 334, 1415–1420. 10.1126/science.1210125 22052975

[B40] Takahashi H. Craig A. M. (2013). Protein tyrosine phosphatases PTPdelta, PTPsigma, and LAR: presynaptic hubs for synapse organization. Trends Neurosci. 36, 522–534. 10.1016/j.tins.2013.06.002 23835198 PMC3789601

[B43] Um S. M. Ha S. Lee H. Kim J. Kim K. Shin W.. (2018). NGL-2 deletion leads to autistic-like behaviors responsive to NMDAR modulation. Cell Rep. 23, 3839–3851. 10.1016/j.celrep.2018.05.087 29949768

[B41] Um J. W. Ko J. (2013). LAR-RPTPs: synaptic adhesion molecules that shape synapse development. Trends Cell Biol. 23, 465–475. 10.1016/j.tcb.2013.07.004 23916315

[B42] Um J. W. Ko J. (2017). Neural glycosylphosphatidylinositol-anchored proteins in synaptic specification. Trends Cell Biol. 27, 931–945. 10.1016/j.tcb.2017.06.007 28743494

[B44] Valnegri P. Sala C. Passafaro M. (2012). Synaptic dysfunction and intellectual disability. Adv. Exp. Med. Biol. 970, 433–449. 10.1007/978-3-7091-0932-8_19 22351067

[B45] Woo J. Kwon S. K. Choi S. Kim S. Lee J. Dunah A. W.. (2009a). Trans-synaptic adhesion between NGL-3 and LAR regulates the formation of excitatory synapses. Nat. Neurosci. 12, 428–437. 10.1038/nn.2279 19252495

[B46] Woo J. Kwon S. K. Kim E. (2009b). The NGL family of leucine-rich repeat-containing synaptic adhesion molecules. Mol. Cell. Neurosci. 42, 1–10. 10.1016/j.mcn.2009.05.008 19467332

[B47] Yin Y. Miner J. H. Sanes J. R. (2002). Laminets: laminin- and netrin-related genes expressed in distinct neuronal subsets. Mol. Cell. Neurosci. 19, 344–358. 10.1006/mcne.2001.1089 11906208

[B48] Yuzaki M. (2011). Cbln1 and its family proteins in synapse formation and maintenance. Curr. Opin. Neurobiol. 21, 215–220. 10.1016/j.conb.2011.01.010 21342763

[B49] Zhang Q. Goto H. Akiyoshi-Nishimura S. Prosselkov P. Sano C. Matsukawa H.. (2016). Diversification of behavior and postsynaptic properties by netrin-G presynaptic adhesion family proteins. Mol. Brain 9:6. 10.1186/s13041-016-0187-5 26746425 PMC4706652

[B50] Zhang Q. Wang J. Fan S. Wang L. Cao L. Tang K.. (2005). Expression and functional characterization of LRRC4, a novel brain-specific member of the LRR superfamily. FEBS Lett. 579, 3674–3682. 10.1016/j.febslet.2005.05.058 15967442

[B51] Zucker R. S. Regehr W. G. (2002). Short-term synaptic plasticity. Annu. Rev. Physiol. 64, 355–405. 10.1146/annurev.physiol.64.092501.114547 11826273

